# Supplementary material for: Autophagy within the mushroom body protects from synapse aging in a non-cell autonomous manner
Source: Nat Commun. 2019 Mar 21;10:1318. doi: 10.1038/s41467-019-09262-2 (PMC6428838; doi:10.1038/s41467-019-09262-2)
Supplement: Supplementary file 1 — Supplementary Information [file 41467_2019_9262_MOESM1_ESM.pdf]

Supplementary Information

Autophagy within the mushroom body learning center protects from synapse aging in a non-cell autonomous manner.

Bhukel *et. al.* 2019

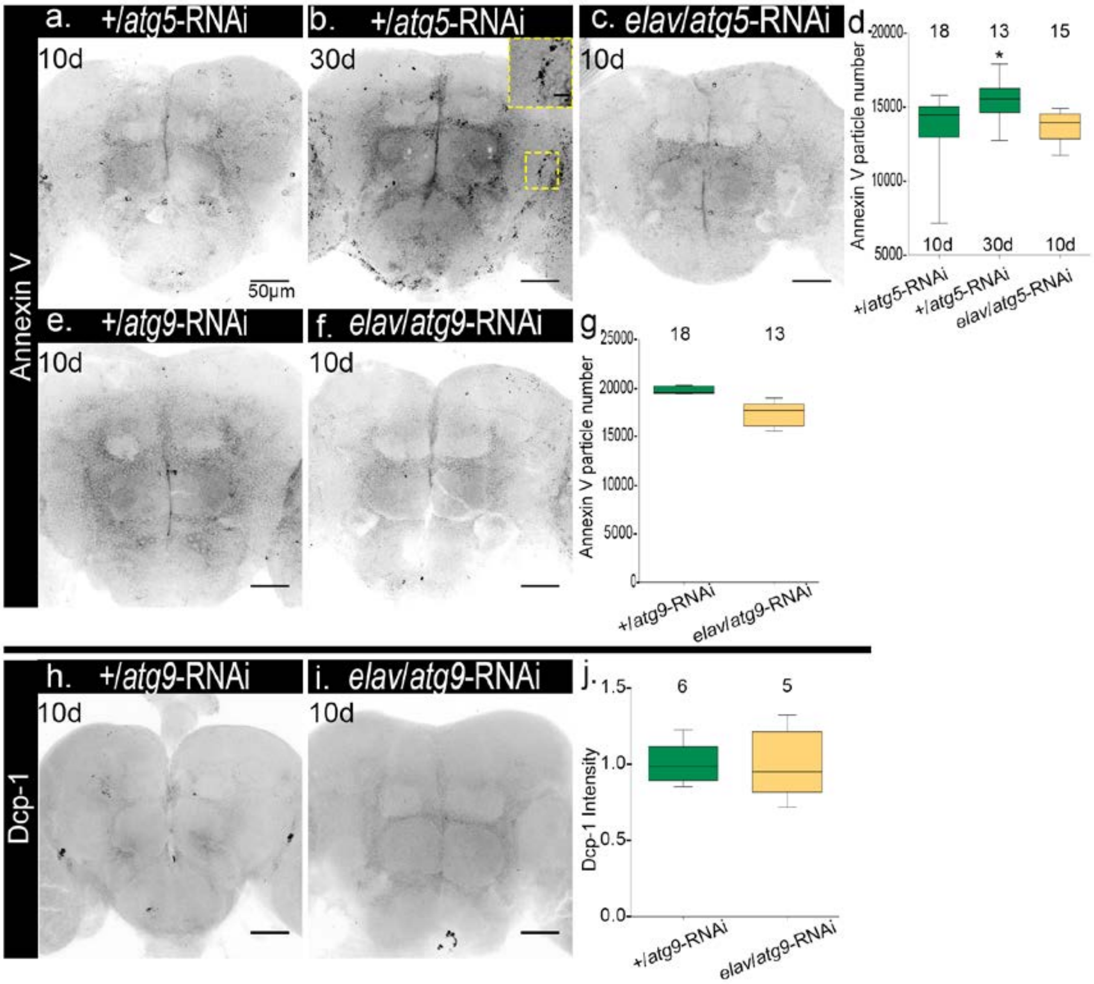

Supplementary Figure 1: Neuronal inhibition of autophagy does not induce apoptosis. Adult brain (a) 10d, +/*atg5*-RNAi, (b) 30d, +/*atg5*-RNAi and (c) 10d, *elav*/*atg5*-RNAi immunostained for Annexin V. Scale bar: $50\mu m$. (d) Quantification of Annexin V particle number within the central brain region. (n = 13-18 independent brains; *p<0.05; $^{ns}p>0.5$, Mann-Whitney U-test with Bon-feroni correction). Adult brain, 10d (e) +/*atg9*-RNAi and (f) *elav*/*atg9*-RNAi immunostained for Annexin V. Scale bar: $50\mu m$. (g) Quantification of Annexin V particle number within the central brain region. (n = 13-18 independent brains; $^{ns}p>0.5$, Mann-Whitney U-test). Adult brain, 10d (h) +/*atg9*-RNAi and (i) *elav*/*atg9*-RNAi immunostained for Dcp-1. Scale bar: $50\mu m$. (j) Quantification of Dcp-1 intensity within the central brain region. (n = 5-6 independent brains; $^{ns}p>0.5$, Mann-Whitney U-test). In the box and whisker plots, the middle line of a box represents the median (50th percentile) and the terminal lines of a box represent the 25th and 75th percentile. The whiskers represent the lowest and the highest value.

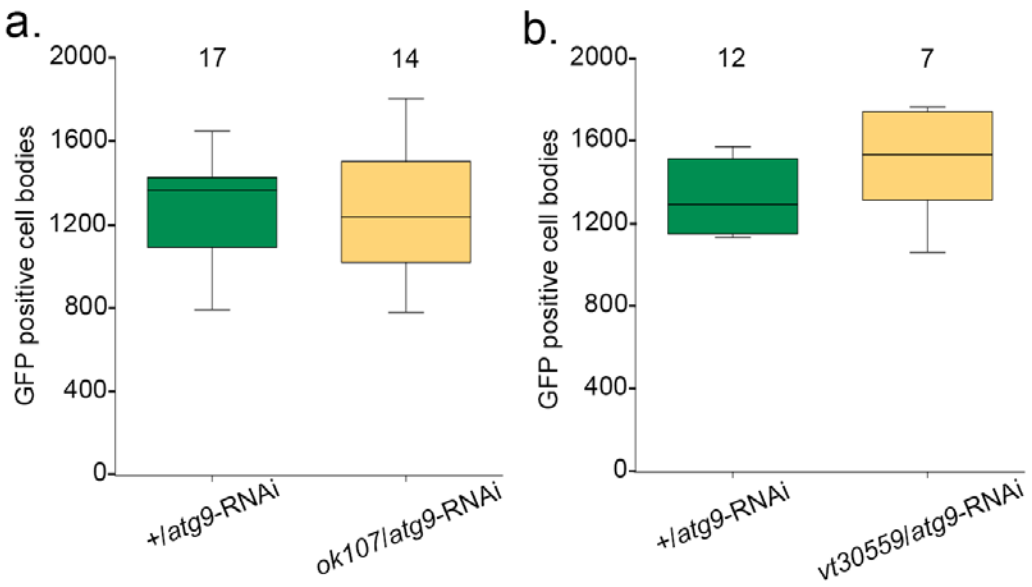

Supplementary Figure 2: Neuronal inhibition of autophagy does not alter cell body counts. Count of GFP positive cell bodies does not differ significantly between (a) +/*atg9*-RNAi (1365) and *ok107*/*atg9*-RNAi (1238), and (b) +/*atg9*-RNAi (1294) and *vt30559*/*atg9*-RNAi (1533). GFP was expressed using the MB driver *ok107*-Gal4 and *vt30559*-Gal4, respectively. GFP positive cell bodies were manually counted in random focal planes with non-overlapping cells and compared between genotypes. No significant difference was found between median cell body counts: +/*atg9*-RNAi (1365), *ok107*/*atg9*-RNAi (1238), +/*atg9*-RNAi (1294) and *vt30559*/*atg9*-RNAi (1533) (n = 7-17; $^{ns}p>0.05$, Mann-Whitney U-test). In the box and whisker plots, the middle line of a box represents the median (50th percentile) and the terminal lines of a box represent the 25th and 75th percentile. The whiskers represent the lowest and the highest value.

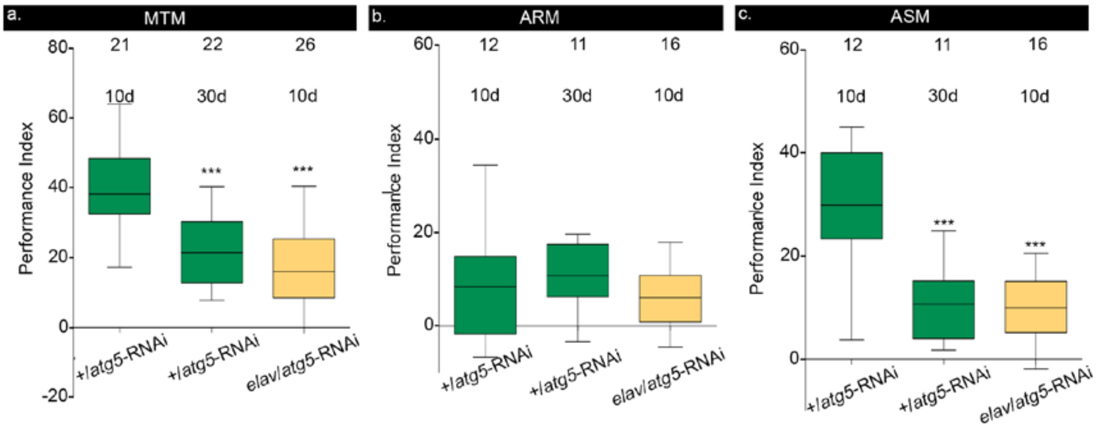

Supplementary Figure 3: Neuronal inhibition of autophagy leads to "early" impairment of age-sensitive olfactory memory. (a) Mid-term memory (MTM) of +/*atg5*-RNAi declined significantly at 30d of age compared to 10d of age. 10d old *elav*/*atg5*-RNAi displayed a similar decrease in their 1h MTM performance (n = 21-26; ***p<0.001, Mann-Whitney U-test with Bon-ferroni correction). (b) Anesthesia-resistant memory (ARM) and (c) Anesthesia-sensitive memory (ASM) of +/*atg5*-RNAi at 10d and 30d of age compared to 10d old *elav*/*atg5*-RNAi (n = 11-16; ***p<0.001; ^{ns}p>0.5, Mann-Whitney U-test with Bon-ferroni correction). In the box and whisker plots, the middle line of a box represents the median (50th percentile) and the terminal lines of a box represent the 25th and 75th percentile. The whiskers represent the lowest and the highest value.

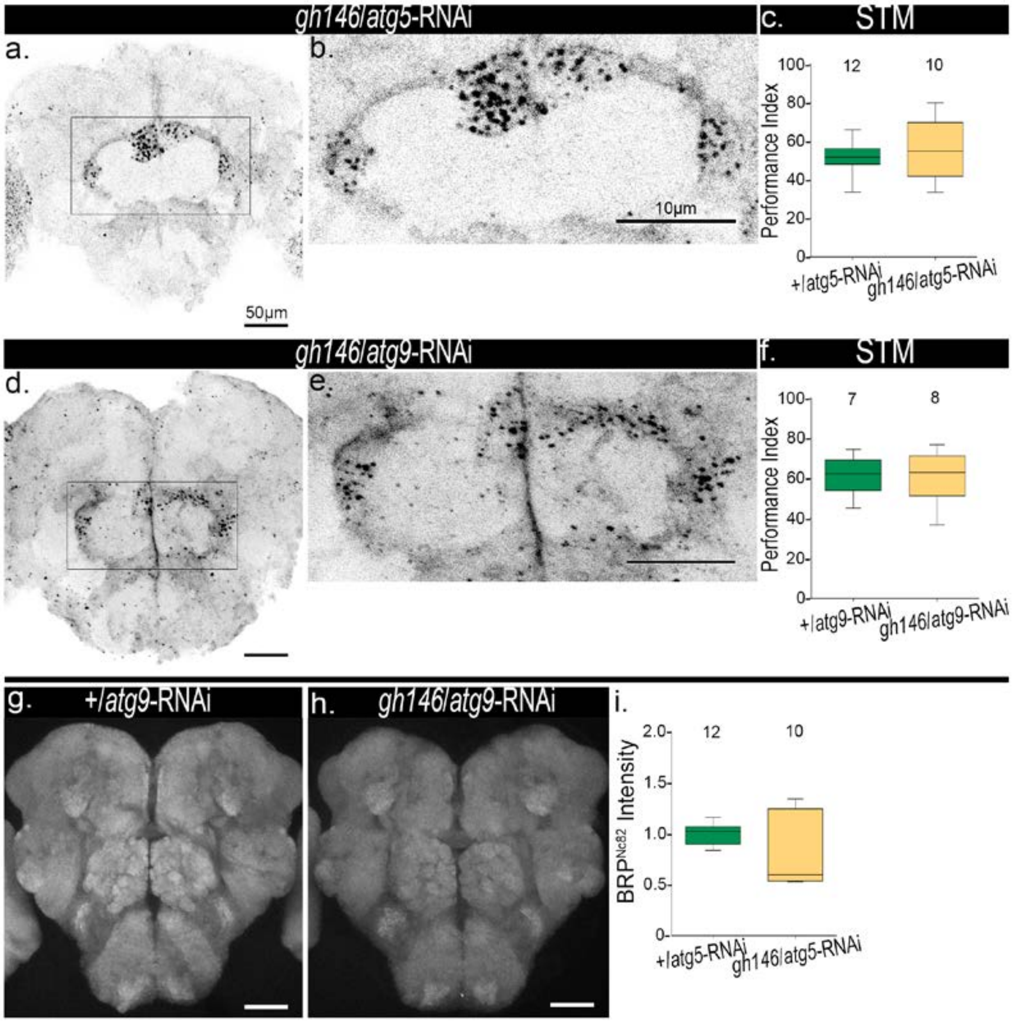

Supplementary Figure 4: Projection neuron-specific suppression of autophagy does not alter Bruchpilot. (a) Immunostainings for p62/Ref(2)p in 10d old adult fly brain *gh146*/*atg5*-RNAi confirms buildup of p62 aggregates in targeted neuron populations. Scale bar: 50μm. (b) Enlarged image of region of interest to show the p62 aggregates. Scale bar: 10um. (c) STM of *gh146*/*atg5*-RNAi compared to +/*atg5*-RNAi (n = 10-12; $^{ns}p>0.5$, Mann Whitney U-test). (d) Immunostainings for p62/Ref(2)p in 10d old adult fly brain *gh146*/*atg9*-RNAi confirms buildup of p62 aggregates in targeted neuron populations. Scale bar: 50μm. (e) Enlarged image of region of interest to show the p62 aggregates. Scale bar: 10μm. (f) STM of *gh146*/*atg9*-RNAi compared to +/*atg9*-RNAi (n = 7-8; $^{ns}p>0.5$, Mann-Whitney U-test). Adult brain, 10d (g) +/*atg9*-RNAi and (h) *gh146*/*atg9*-RNAi immunostained for BRP^{Nc82}. Scale bar: 50μm. (i) Quantification of BRP^{Nc82} intensity within the central brain region normalized to control (n = 6-11 independent brains; $^{ns}p>0.5$, Mann-Whitney U-test). In the box and whisker plots, the middle line of a box represents the median (50th percentile) and the terminal lines of a box

represent the 25th and 75th percentile. The whiskers represent the lowest and the highest value.

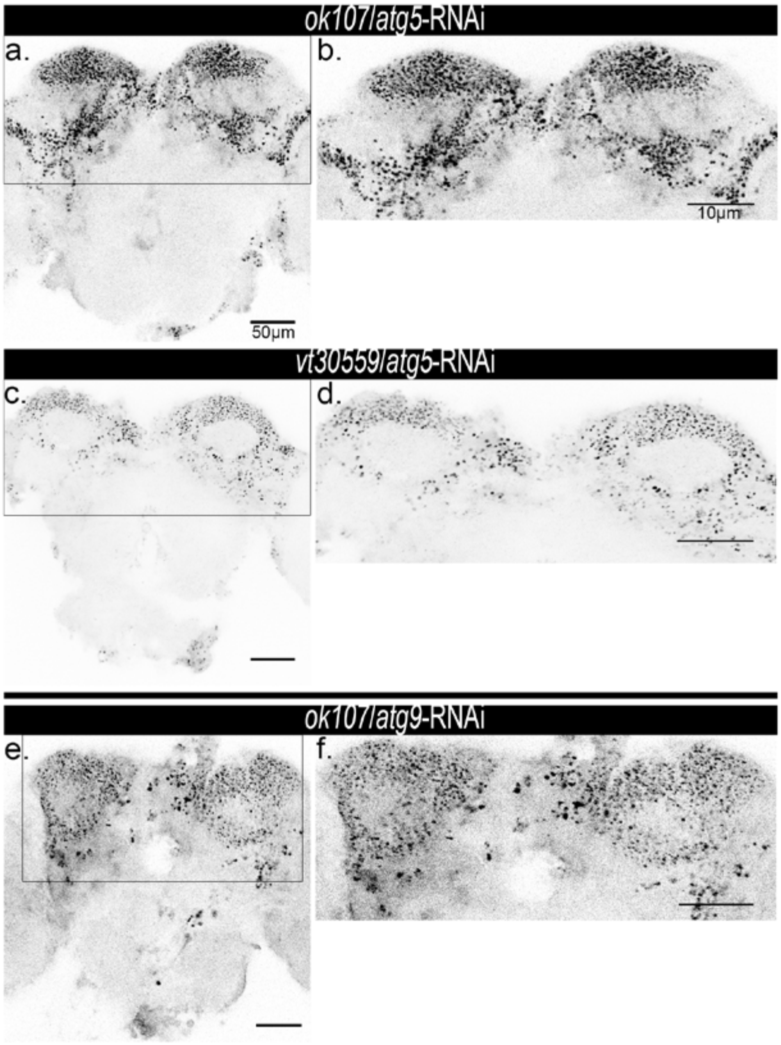

Supplementary Figure 5: Mushroom body-specific suppression of autophagy causes cell Autonomous aggregation of p62. (a) Immunostainings for p62/Ref(2)p in 10d old adult fly brain *ok107*/*atg5*-RNAi confirms buildup of p62 aggregates in targeted neuron populations. Scale bar: 50μm. (b) Enlarged image of region of interest to show the p62 aggregates in Kenyon cell bodies. Scale bar: 10μm. (c) Immunostainings for p62/Ref(2)p in 10d old adult fly brain *vt30559*/*atg5*-RNAi confirms buildup of p62 aggregates in targeted neuron populations. Scale bar: 50μm. (d) Enlarged image of region of interest to show the p62 aggregates in Kenyon cell bodies. Scale bar: 10μm. (e) Immunostainings for p62/Ref(2)p in 10d old adult fly brain *ok107*/*atg9*-RNAi confirms buildup of p62 aggregates in targeted neuron populations. Scale bar: 50μm. (f) Enlarged image of region of interest to show the p62 aggregates in Kenyon cell bodies. Scale bar: 10μm.

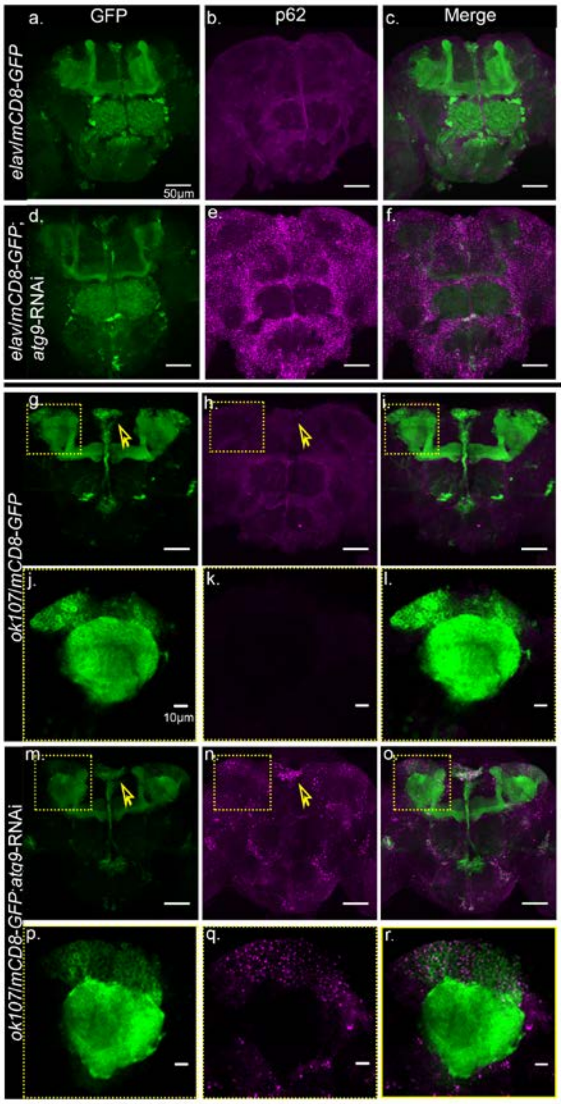

Supplementary Figure 6: Gal4-driven targeted suppression of autophagy in specific neuron populations. (a-c) Immunostainings for GFP in 10d old adult fly brain *elav/mCD8-GFP* confirms pan-neuronal expression of elav-Gal4. p62 stainings indicate basal levels of autophagy (z-projection). Scale bar: 50μm. (d-f) Immunostainings for p62 confirm brain-wide knockdown of autophagy in *elav/mCD8-GFP*;*atg9*-RNAi (z-projection). Scale bar: 50μm. (g-i) Immunostainings for GFP in 10d old adult fly brain *ok107/mCD8-GFP* confirms mushroom body targeted expression of driver line *ok107*-Gal4. Besides a strong expression in mushroom body (yellow box), the driver line has expression in some other neuron populations (see arrow). p62 stainings indicate basal levels of autophagy (z-projection). (j-l) Blow-up of Kenyon cell bodies (single z-plane). Scale bar: 10 μm. (m-o) Immunostainings for p62 confirm knockdown of autophagy in expression domain of *ok107*-Gal4 (z-projection). (p-r) Blow-up of Kenyon cell bodies (single z-plane). p62 agreegates accumulate in Kenyon cell bodies. Scale bar: 10μm.

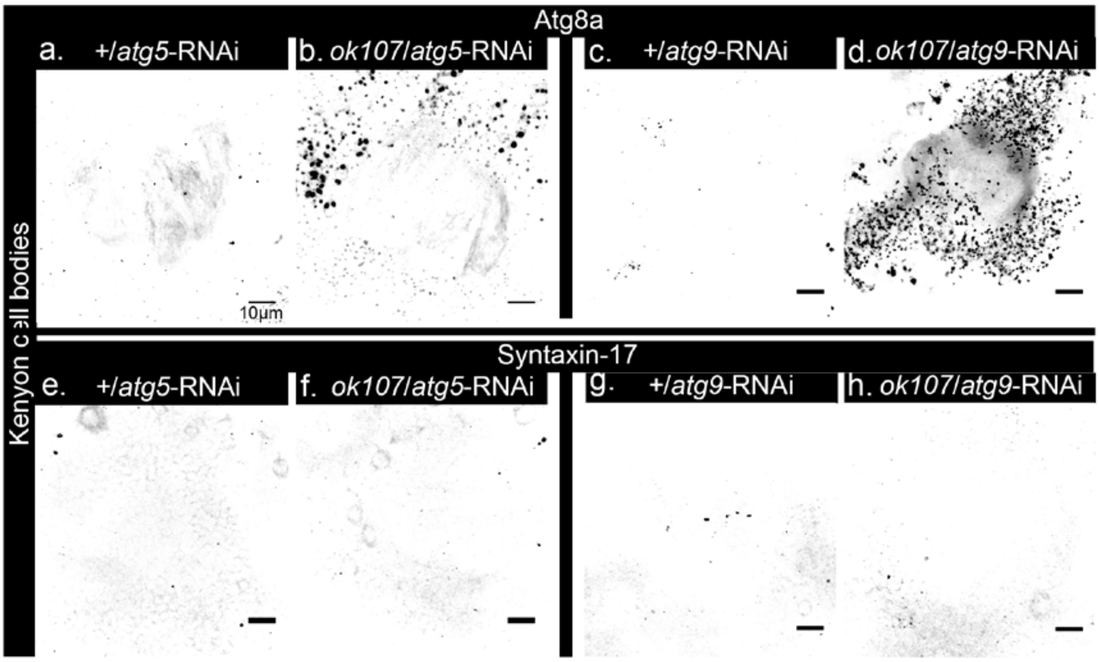

Supplementary Figure 7: Mushroom body-specific suppression of autophagy causes aggregation of Atg8a but not Syntaxin-17. (a-b) Knockdown of *atg5* in mushroom bodies induce Atg8a aggregation in Kenyon cell bodies. Scale bar: 10 μm. (c-d) Knockdown of *atg9* in mushroom bodies induce Atg8a aggregation in Kenyon cell bodies. Scale bar: 10 μm. Mushroom body specific knockdown of either (e-f) *atg5* or (g-h) *atg9* did not affect Syntaxin-17 levels in Kenyon cell bodies. Scale bar: 10 μm.

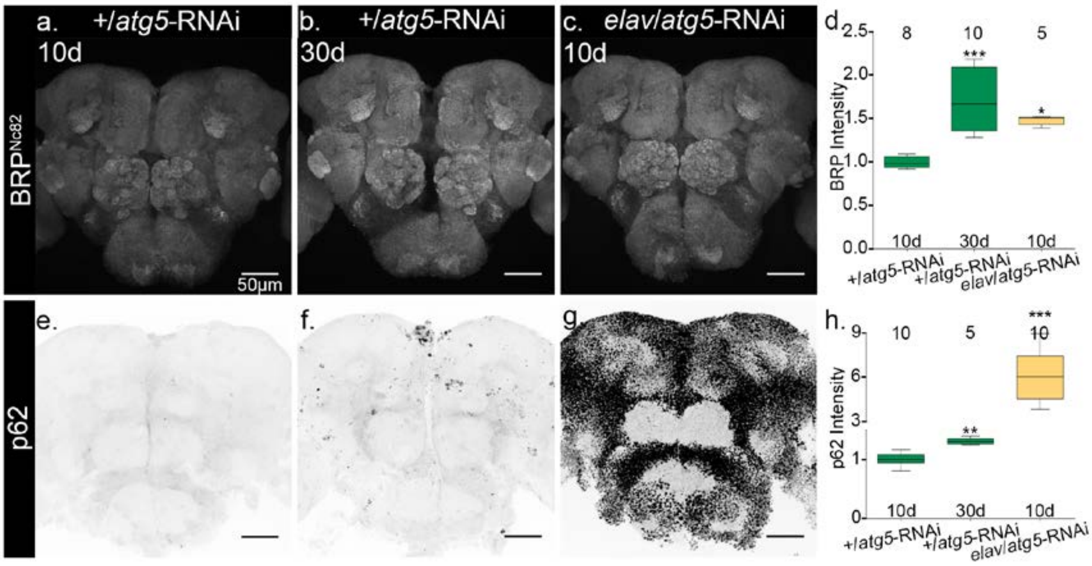

Supplementary Figure 8: Neuronal inhibition of autophagy induces an early increase in Bruchpilot in young flies. Adult brain (a) 10d, +/*atg5*-RNAi, (b) 30d, +/*atg5*-RNAi and (c) 10d, *elav*/*atg5*-RNAi immunostained for BRP^{Nc82}. Scale bar: 50 μm. (d) Quantification of BRP intensity within the central brain region normalized to control (n = 5-10 independent brains; *p<0.05; ***p<0.001; ^{ns}p>0.5; Mann-Whitney U-test). Adult brain, (e) 10d, +/*atg5*-RNAi, (f) 30d, +/*atg5*-RNAi and (g) 10d, *elav*/*atg5*-RNAi, immunostained for p62. Scale bar: 50 μm. (h) Quantification of p62 intensity within the central brain region normalized to control (n = 5-10 independent brains; **p<0.01; ***p<0.001; ^{ns}p>0.5; Mann-Whitney U-test). In the box and whisker plots, the middle line of a box represents the median (50th percentile) and the terminal lines of a box represent the 25th and 75th percentile. The whiskers represent the lowest and the highest value.

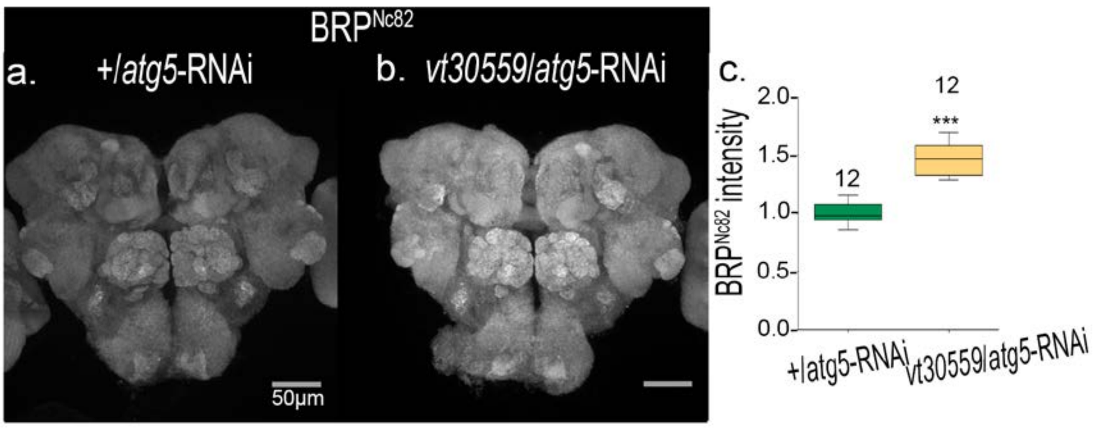

Supplementary Figure 9: Mushroom body-specific attenuation of autophagy induces brain-wide increase in Bruchpilot. Adult brain (a) +/*atg5*-RNAi and (b) *vt30559*/*atg5*-RNAi immunostained for BRP^{Nc82}. Scale bar: 50 μm. (c) Quantification of BRP^{Nc82} intensity within the central brain region normalized to control (n = 12 independent brains; ***p<0.001, Mann-Whitney U-test). In the box and whisker plots, the middle line of a box represents the median (50th percentile) and the terminal lines of a box represent the 25th and 75th percentile. The whiskers represent the lowest and the highest value.

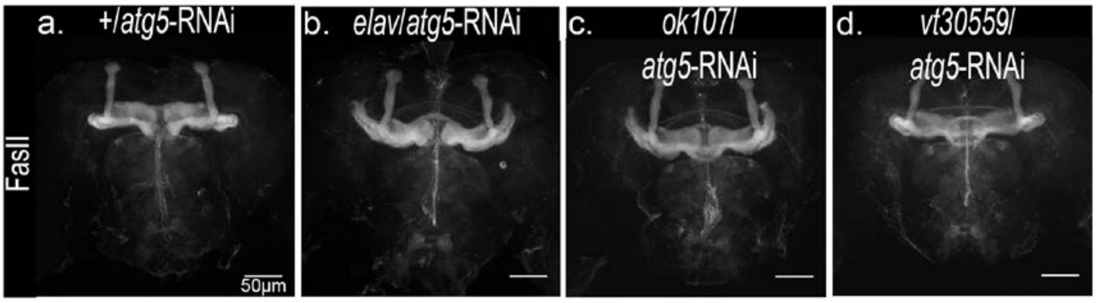

Supplementary Figure 10: Mushroom body specific attenuation of autophagy does not cause any defects in their development. Adult brain (a) +/*atg5*-RNAi, (b) *elav*/*atg5*-RNAi, (c) *ok107*/*atg5*-RNAi and (d) *vt30559*/*atg5*-RNAi immunostained for FasII. Scale bar: 50 μm.

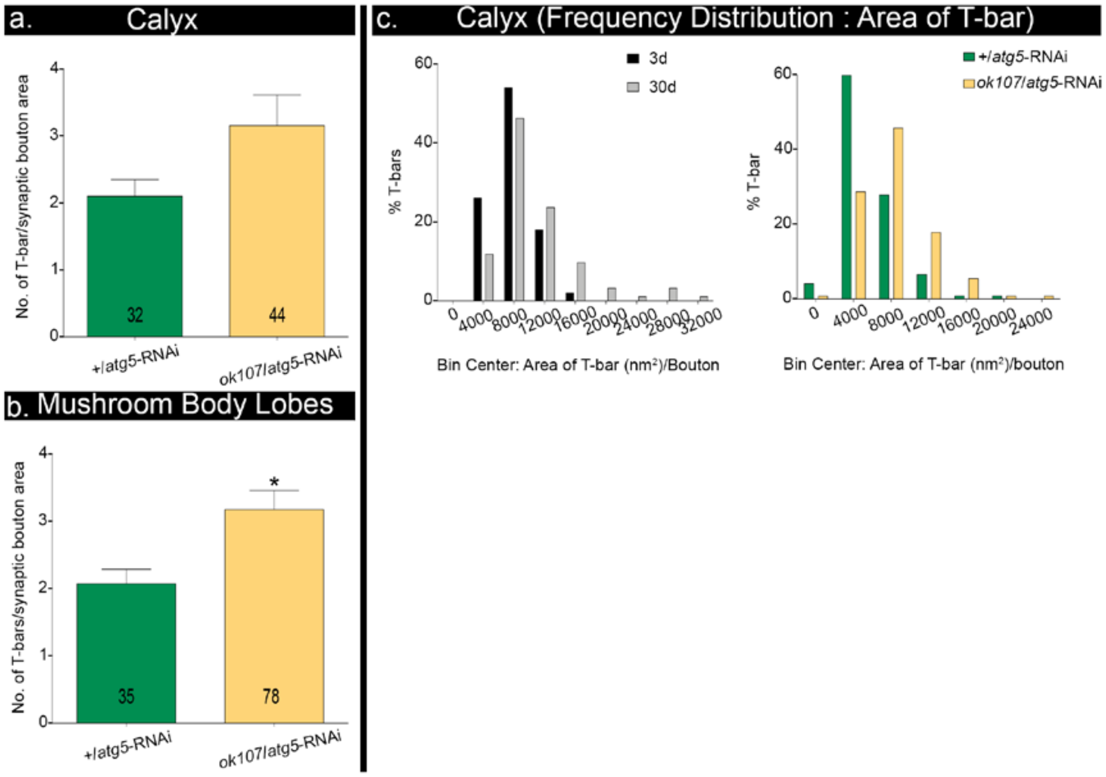

Supplementary Figure 11: Mushroom body-specific attenuation of autophagy causes non-cell autonomous increase in active zone density. Number of T-bars per synaptic bouton area increase in (a) Calyx and (b) Mushroom body lobes upon mushroom body specific attenuation of *atg9*. (n = 32-78 synaptic boutons; 3-5 independent animals; *$p<0.05$; $^{ns}p>0.5$; Mann Whitney U-test). At calyx, +/*atg9*-RNAi: 2.099 ± 0.2490 (Mean ± SEM), *ok107*/*atg9*-RNAi: 3.151 ± 0.4632. At MB lobes, +/*atg9*-RNAi: 2.069 ± 0.2145, *ok107*/*atg9*-RNAi: 3.171 ± 0.2848. Distribution of T-bar area shifted towards a bigger size in (c) 30d w1118 compared to 3d w1118 and in (d) *ok107*/*atg9*-RNAi compared to +/*atg9*-RNAi in Calyx.

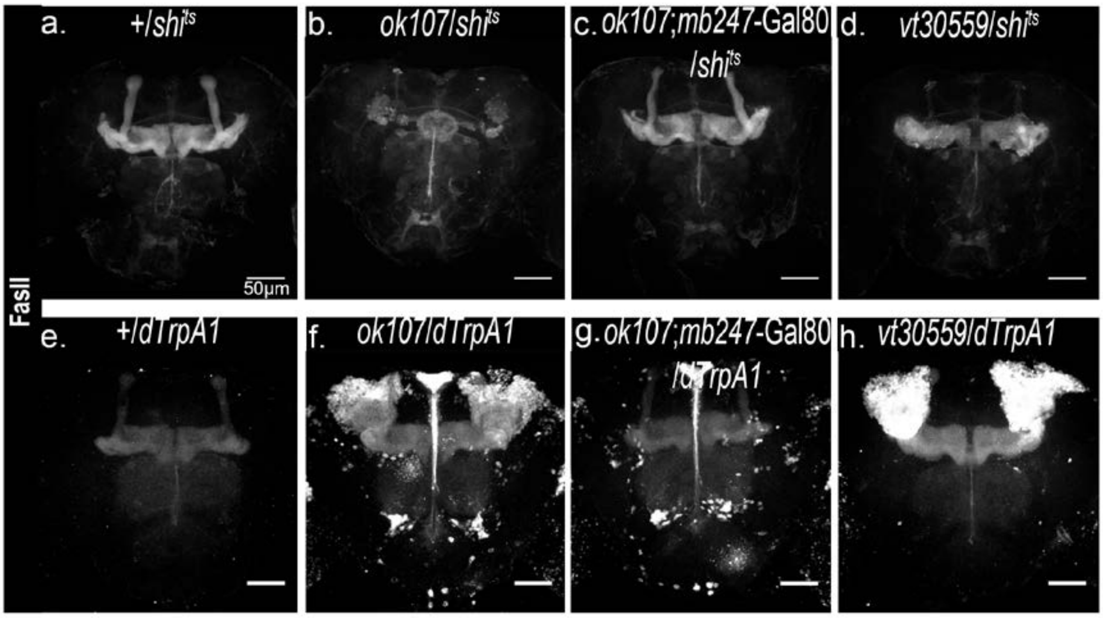

Supplementary Figure 12: Modulation of mushroom body neuron activity causes developmental defects in mushroom body. Adult brain, 10d (a) +/*shi*^{ts}, (b) *ok107*/*shi*^{ts} (c) *ok107*;*mb247*-Gal80/*shi*^{ts}, (d) *vt30559*/*shi*^{ts}, (e) +/*dTrpA1*, (f) *ok107*/*dTrpA1*, (g) *ok107*;*mb247*-Gal80/*dTrpA1* and (h) *vt30559*/*dTrpA1* immunostained for FasII. Scale bar: 50μm.

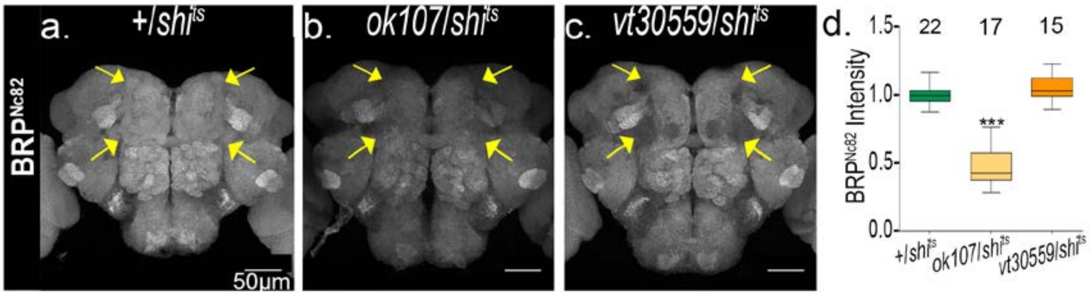

Supplementary Figure 13: Loss of mushroom body neuron activity does not induce presynaptic metaplasticity. Adult brain, 10d, (a) +/*shi*^{ts}, (b) *ok107/shi*^{ts} and (c) *vt30559/shi*^{ts} immunostained for BRP^{Nc82}. Scale bar: 50μm. (d) Quantification of BRP^{Nc82} intensity within the central brain region normalized to +/*shi*^{ts} (n = 15-22 independent brains; ***p<0.001; One-way ANOVA; Kruskal-Walis test). In the box and whisker plots, the middle line of a box represents the median (50th percentile) and the terminal lines of a box represent the 25th and 75th percentile. The whiskers represent the lowest and the highest value.

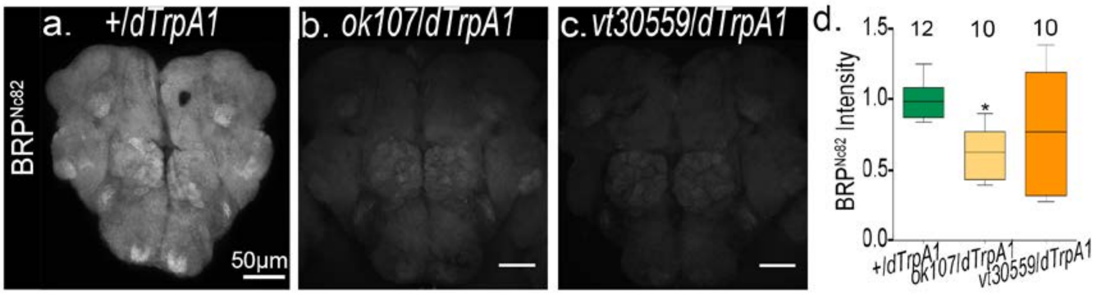

Supplementary Figure 14: Gain of mushroom body neuron activity does not induce presynaptic metaplasticity. Adult brain, 10d, (a) +/*dTrpA1*, (b) *ok107*/*dTrpA1* and (c) *vt30559*/*dTrpA1* immunostained for BRP^{Nc82}. Scale bar: $50\mu m$. (d) Quantification of BRP^{Nc82} intensity within the central brain region normalized to +/*dTrpA1* (n = 15-22 independent brains; *$p<0.05$; One-way ANOVA; Kruskal-Walis test). In the box and whisker plots, the middle line of a box represents the median (50th percentile) and the terminal lines of a box represent the 25th and 75th percentile. The whiskers represent the lowest and the highest value.

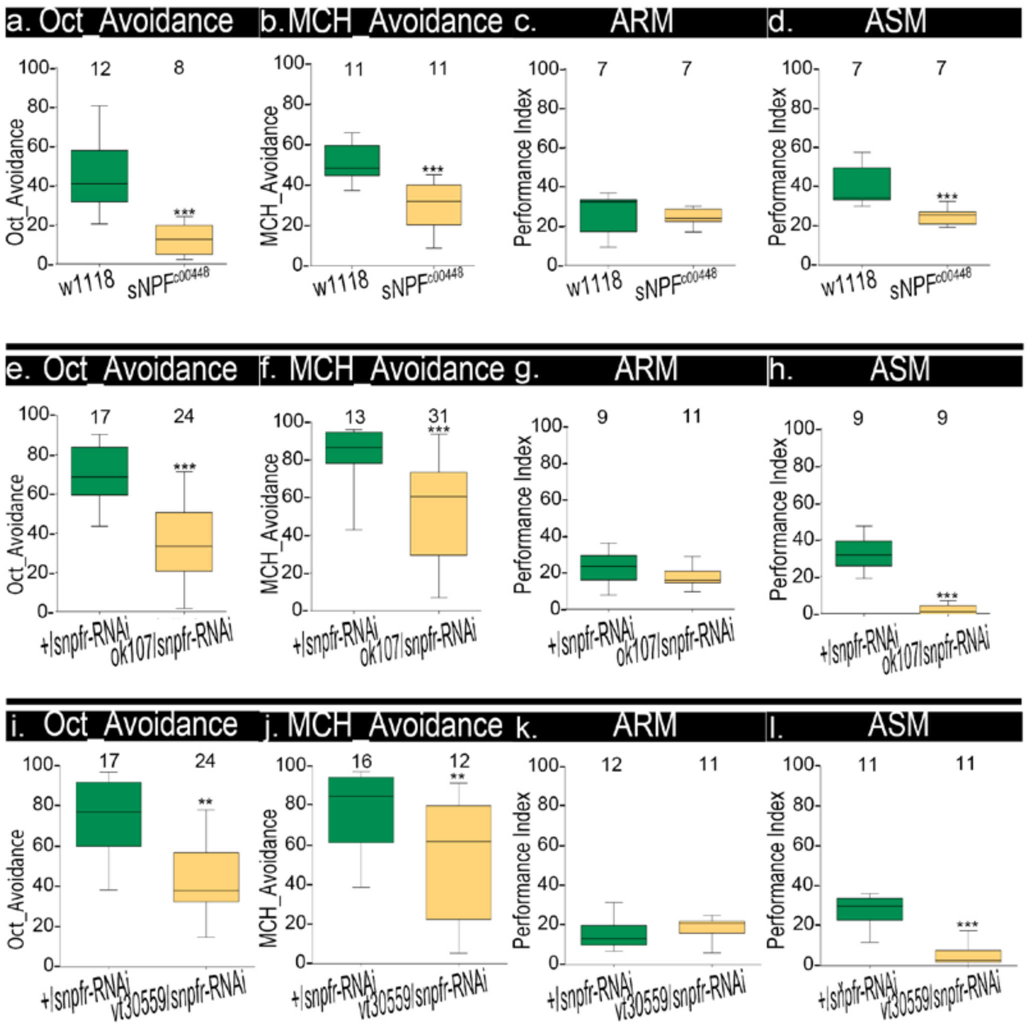

Supplementary Figure 15: sNPF signaling is required for odor avoidance in *Drosophila*. (a) Octanol avoidance of w1118 and *sNPF*^{c00448} (n = 8-12; ***p<0.001; Mann-Whitney U-test). (b) MCH avoidance of w1118 and *sNPF*^{c00448} (n = 11; ***p<0.001; Mann-Whitney U-test). (c) ARM and (d) ASM of w1118 and *sNPF*^{c00448} (n = 7; ***p<0.001; ^{ns}p>0.5; Mann-Whitney U-test). (e) Octanol avoidance of +/*snpfr*-RNAi and *ok107/snpfr*-RNAi (n = 17-24; ***p<0.001; Mann-Whitney U-test). (f) MCH avoidance of +/*snpfr*-RNAi and *ok107/snpfr*-RNAi (n = 13-31; ***p<0.001; Mann-Whitney U-test). (g) ARM and (h) ASM of +/*snpfr*-RNAi and *ok107/snpfr*-RNAi (n = 9-11; ***p<0.001; ^{ns}p>0.5; Mann-Whitney U-test). (i) Octanol avoidance of +/*snpfr*-RNAi and *vt30559/snpfr*-RNAi (n = 17-24; **p<0.01; Mann-Whitney U-test). (j) MCH avoidance of +/*snpfr*-RNAi and *vt30559/snpfr*-RNAi (n = 12-16; **p<0.01; Mann-Whitney U-test). (k) ARM and (l) ASM of +/*snpfr*-RNAi and *vt30559/snpfr*-RNAi (n = 11-12; ***p<0.001; ^{ns}p>0.5; Mann-Whitney U-test). In the box and whisker plots, the middle line of a box represents the median (50th percentile) and the terminal lines of a box represent the 25th and 75th percentile. The whiskers represent the lowest and the highest value.

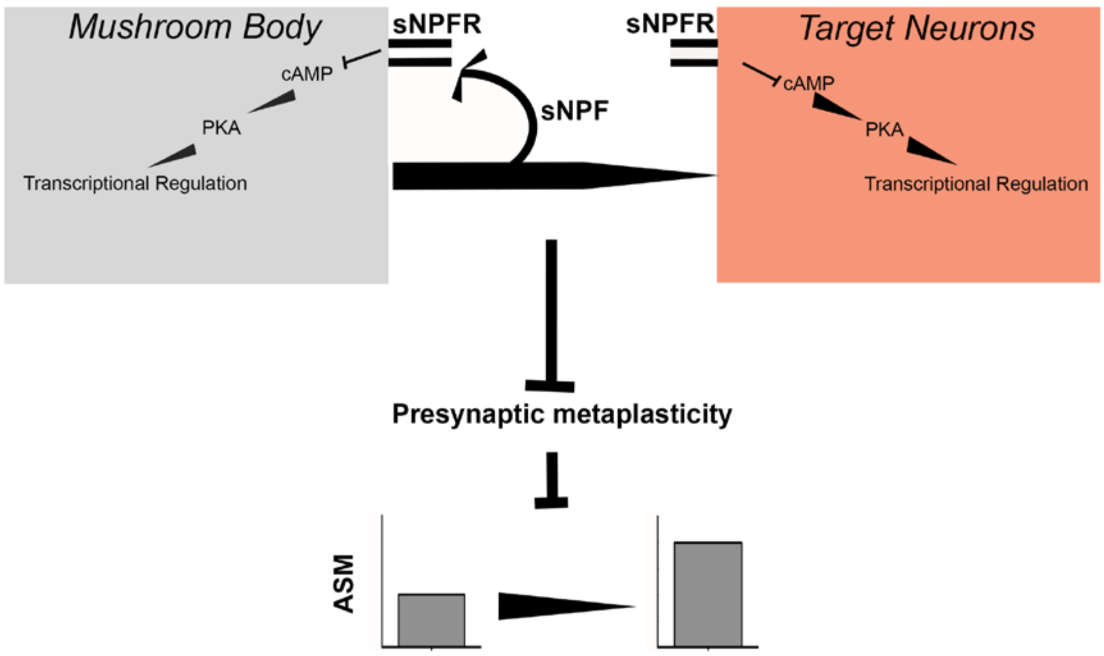

Supplementary Figure 16: Model. The mushroom bodies integrate the metabolic state of the flies via cross talk between autophagy and sNPF signaling with the decision whether to form memories or not. A block in this cross talk with aging gives rise to presynaptic metaplasticity, which initiates the age-induced memory impairment in *Drosophila*.

Gene Name	+/RNAi p62 aggregation	elav/RNAi p62 aggregation
atg1	-	-
atg17	-	+
atg9	-	+++
atg7	-	-
atg5	-	+++
atg8a	-	+
syx-17	-	-

Supplementary Table 1: *atg*-genes subjected to RNA interference mediated knockdown. RNAi knockdown lines: *atg1*, *atg17*, *atg9*, *atg7*, *atg5*, *atg8a* and *syx-17* were screened for p62/Ref(2)p accumulation. Of all the tested candidates, suppression of *atg17*, *atg9*, *atg5* and *atg8a* cause p62 aggregation. $^{-}$No p62 aggregation, $^{+}$Low p62 aggregation, $^{+++}$high p62 aggregation

Genotype	Octanol	3-Methylcyclohexanol
+/*atg5*-RNAi	23.92±3.392	37.63±6.438
elav/*atg5*-RNAi	26.07±4.012	20.26±5.043
+/*atg9*-RNAi	25.56±3.917	55.71±8.466
elav/*atg9*-RNAi	33.16±5.538	29.60±7.695
+/*atg5*-RNAi	23.92±3.392	37.63±6.438
ok107/*atg5*-RNAi	24.74±3.363	30.74±5.113
+/*atg5*-RNAi	48.73±3.9	58.52±5.168
vt30559/*atg5*-RNAi	49.27±10.36	55.52±8.406
+/*atg9*-RNAi	25.56±3.917	55.71±8.466
ok107/*atg9*-RNAi	24.55±3.656	50.61±8.08
+/*atg5*-RNAi	47.98±7.848	51.31±6.652
+/*ok107*;*mb247*	53.74±4.572	61.67±4.24
ok107;*mb247*/*atg5*-RNAi	50.16±5.823	42.64±4.3
+/*atg5*-RNAi	55.32±6.214	37.34±5.653
gh146/*atg5*-RNAi	36.32±4.090	35.33±5.685
+/*atg9*-RNAi	29.17±4.304	46.35±5.897
gh146/*atg9*-RNAi	25.63±5.609	32.22±3.801

Supplementary Table 2: Aversive olfactory Avoidance in different genotypes used in this study. None of the experimental groups show significantly impaired aversion to the odors used in this study compared to their corresponding control. Values are represented as Mean ± SEM.
