# Supplementary material for: Autophagy within the mushroom body protects from synapse aging in a non-cell autonomous manner
Source: Nat Commun. 2019 Mar 21;10:1318. doi: 10.1038/s41467-019-09262-2 (PMC6428838; doi:10.1038/s41467-019-09262-2)
Supplement: Supplementary file 3 — Reporting Summary [file 41467_2019_9262_MOESM3_ESM.pdf]

nature research

Corresponding author(s): Stephan J. Sigrist

# Reporting Summary

Nature Research wishes to improve the reproducibility of the work that we publish. This form provides structure for consistency and transparency in reporting. For further information on Nature Research policies, see Authors & Referees and the Editorial Policy Checklist.

## Statistical parameters

When statistical analyses are reported, confirm that the following items are present in the relevant location (e.g. figure legend, table legend, main text, or Methods section).

| n/a | Confirmed | |
|---|---|---|
| ☐ | ☒ | The exact sample size (*n*) for each experimental group/condition, given as a discrete number and unit of measurement |
| ☐ | ☒ | An indication of whether measurements were taken from distinct samples or whether the same sample was measured repeatedly |
| ☐ | ☒ | The statistical test(s) used AND whether they are one- or two-sided<br>*Only common tests should be described solely by name; describe more complex techniques in the Methods section.* |
| ☐ | ☒ | A description of all covariates tested |
| ☐ | ☒ | A description of any assumptions or corrections, such as tests of normality and adjustment for multiple comparisons |
| ☐ | ☒ | A full description of the statistics including central tendency (e.g. means) or other basic estimates (e.g. regression coefficient) AND variation (e.g. standard deviation) or associated estimates of uncertainty (e.g. confidence intervals) |
| ☐ | ☒ | For null hypothesis testing, the test statistic (e.g. *F*, *t*, *r*) with confidence intervals, effect sizes, degrees of freedom and *P* value noted<br>*Give P values as exact values whenever suitable.* |
| ☒ | ☐ | For Bayesian analysis, information on the choice of priors and Markov chain Monte Carlo settings |
| ☒ | ☐ | For hierarchical and complex designs, identification of the appropriate level for tests and full reporting of outcomes |
| ☒ | ☐ | Estimates of effect sizes (e.g. Cohen's *d*, Pearson's *r*), indicating how they were calculated |
| ☐ | ☒ | Clearly defined error bars<br>*State explicitly what error bars represent (e.g. SD, SE, CI)* |

*Our web collection on statistics for biologists may be useful.*

## Software and code

Policy information about availability of computer code

| | |
|---|---|
| Data collection | Leica LAS X |
| Data analysis | Amira 5.0, FiJi, GraphPad Prism 5, |

For manuscripts utilizing custom algorithms or software that are central to the research but not yet described in published literature, software must be made available to editors/reviewers upon request. We strongly encourage code deposition in a community repository (e.g. GitHub). See the Nature Research guidelines for submitting code & software for further information.

## Data

Policy information about availability of data

All manuscripts must include a data availability statement. This statement should provide the following information, where applicable:

- Accession codes, unique identifiers, or web links for publicly available datasets
- A list of figures that have associated raw data
- A description of any restrictions on data availability

Data will be available before publication



April 2018

# Field-specific reporting

Please select the best fit for your research. If you are not sure, read the appropriate sections before making your selection.

☒ Life sciences ☐ Behavioural & social sciences ☐ Ecological, evolutionary & environmental sciences

For a reference copy of the document with all sections, see nature.com/authors/policies/ReportingSummary-flat.pdf

# Life sciences study design

All studies must disclose on these points even when the disclosure is negative.

| | |
|---|---|
| Sample size | The sample size chosen throughout this study is same as others in the field. |
| Data exclusions | No data was excluded. |
| Replication | All attempts at replication were successful. |
| Randomization | Different genotypes were compared in this study and each genotype is a group. |
| Blinding | Investigators were blinded during data analysis |

# Reporting for specific materials, systems and methods

## Materials & experimental systems

| n/a | Involved in the study | |
|---|---|---|
| ☒ | ☐ | Unique biological materials |
| ☐ | ☒ | Antibodies |
| ☒ | ☐ | Eukaryotic cell lines |
| ☒ | ☐ | Palaeontology |
| ☐ | ☒ | Animals and other organisms |
| ☒ | ☐ | Human research participants |

## Methods

| n/a | Involved in the study | |
|---|---|---|
| ☒ | ☐ | ChIP-seq |
| ☒ | ☐ | Flow cytometry |
| ☒ | ☐ | MRI-based neuroimaging |

## Antibodies

| | |
|---|---|
| Antibodies used | Mouse BRPNc82 (1:25; DSHB), Mouse FasII1D4 (1:40; DSHB), Rabbit p62 (1:2000; Gabor Juhasz), Rabbit sNPF (1:2000; Jan Veenstra), Mouse GFP (1:1000), Rabit AnnexinV (1:100, Ab14196), Rabbit Dcp-1 (1:100, Asp216), Goat anti Mouse Cy3 (1:500; Ab97035), Goat anti Rabbit Alexa 488(1:500; A11008), Goat anti Mouse aberrior star 635p (1:200; #200020075), Goat anti Rabbit Alexa 594 (1:200; A11037), Mouse Tubulin (1:10000; T9026), Rabbit Atg8a (1:1000; Ab109364), Goat anti Mouse Peroxidase (1:5000; Dianova 115035166) and Goat anti Rabbit Peroxidase (1:5000; Dianova 111035144), |
| Validation | BRPNc82: Immunofluorescence, Immunohistochemistry, Western Blot; Species: Drosophila<br>FASII: ELISA, Immunofluorescence, Immunohistochemistry, Western Blot; Species: Drosophila<br>Tubulin: Indirect Immunofluorescence, Western Blot; Species: Yeast, human, rat, chicken, fungi, amphibian, bovine, mouse<br>sNPF: Immunofluorescence; Species: Drosophila<br>p62: Immunofluorescence, Western Blot; Species: Drosophila<br>AnnexinV: Flow cytometry, Western blot, Immunohistochemisty-FoFr, Immunohistochemisty-Fr, Immunohistochemisty-P, Immunocytochemistry, Immunofluorescence; Species: Mouse, rat, human.<br>Dcp-1: Western Blot, Immunofluorescence; Species: Drosophila<br>Atg8a: Flow cytometry, Western blot, Immunohistochemisty, Immunocytochemistry, Immunofluorescence; Species: Mouse, rat, human, silk worm. |

## Animals and other organisms

Policy information about studies involving animals; ARRIVE guidelines recommended for reporting animal research

| | |
|---|---|
| Laboratory animals | Drosophila melanogaster: w1118, snpfr-RNAi, sNPFc00448, dilp2-Gal4 (#37516), atg7-RNAi (#27707, #34369), atg5-RNAi (#34899, #27551), atg9-RNAi (#34901), atg8-RNAi (#28989), syx17-RNAi (#25896), vt30559-Gal4 (#206077), atg17-RNAi |

(#KK104864), elav-Gal4, appl-Gal4, gh146-Gal4, ok107-Gal4, ok107-Gal4; mb247-Gal80
ok107-Gal4; tub-Gal80ts

Wild animals

The study didnot involve wild animals

Field-collected samples

Study didnot involve samples collected from the field.
